# Ultra culture-ultra reality: a content analysis of YouTube depictions of ultra endurance sport and comparisons to scientific literature

**DOI:** 10.3389/fspor.2023.1192401

**Published:** 2023-07-25

**Authors:** Jill Colangelo, Alexander Smith, Ana Buadze, Michael Liebrenz

**Affiliations:** ^1^ Department of Forensic Psychiatry, University of Bern, Bern, Switzerland; ^2^ Department of Psychiatry, Psychotherapy and Psychosomatics, Psychiatric Hospital, University of Zurich, Zurich, Switzerland

**Keywords:** ultra endurance athletes, ultra endurance sport, sports medicine, sports psychiatry, media, sport culture

## Abstract

**Introduction:**

Interest in ultra endurance sport (UES) is increasing, with relevant events growing in popularity. However, these activities may encompass more complex characteristics and demands that do not correspond to scientifically validated correlations between physical activity and improved health. It is unknown whether high volume training for UES can have adverse implications, although certain sociocultural aspects of western society, such as an emphasis on autonomy and self-directed success, may encourage participation as a representation of personal achievement. As media depictions of UES can be highly influential, we aimed to explore prominent messages to better comprehend the values and aspirations of established and aspiring ultra endurance athletes (UEA).

**Methods:**

Using qualitative, conventional content analysis techniques, we evaluated *n* = 15 popular YouTube videos on various UES to synthesize the most common themes. A YouTube search was conducted, and videos were selected based on sport and relevance. Subsequently, videos were analyzed and coded to discern the most frequently repeated themes.

**Results:**

Five recurring themes were identified: discussion of the mental aspects of UES participation; the adoption of folk-tale storytelling; dietary habits of UEA; a focus on pain and suffering; the use of words associated with mental illness as a colloquial descriptor of UES/UEA.

**Discussion:**

YouTube depictions of UES participation appear to accentuate suffering and co-occurring phenomena as attractive qualities. These resonate with sociocultural ideologies in western contexts, which emphasize fitness and personal success, but appear inconsistent with evidence around potentially adverse health-related implications of UES.

**Conclusion:**

Discrepancies between popular portrayals of UES and scientific evidence suggest a need for greater community education and information sharing about sport-specific risk factors for physical and mental health conditions and appropriate training protocols. Further research is also required to better define the relationship between motivations for UES participation and overall health using interdisciplinary approaches.

## 1. Introduction

Popular awareness is growing about ultra endurance sports (1, 2), which are generally defined as any sport lasting six hours or more (e.g., running, cycling, swimming, rowing, triathlon) (3). Extensive training is typically required to successfully complete races that range from 50 km (as in ultrarunning) to over 160 km in a single day, as well as multi-day events (3, 4). Consequently, training durations and volumes tend to be significant for ultra-endurance athletes (UEA)^1^, though this is contingent on individual speeds and terrain (3, 4). In scientific literature, there is a robust evidence-base illustrating the overall health benefits of exercise (5–7). Contrastingly, to the authors' knowledge, no comparable, established associations have been observed for very high volumes of endurance exercise.

As interest in UES increases, we believe that interdisciplinary approaches are necessary to better understand motivators for participation and physiological and psychological outcomes, particularly as these sports and their athletes may be considered to be the “ne plus ultra” of fitness and health (8, 9). Due to the extreme nature of training and competition, it is likely that individuals engage with UES for reasons extending beyond the pursuit of fitness. In that regard, cultural representations can reflect societal norms and values, providing insights into how UES are more broadly considered and why certain athletes may be drawn to them.

### 1.1. Sociocultural contexts of UES participation

Discussions about UES participation and the corresponding societal drivers underpinning these activities have roots within western culture, which may similarly value concepts of individualism, autonomy, and power as bodily currency (10), among others. Historically, endurance running has transcended conventional definitions of sporting practice, often shifting in meaning and application based on wider paradigms (11). As early endurance running competitions gained recognition in western countries in the late 1800s, relevant discourse centered around notions of discipline, overcoming the self, and perseverance (12). Notably, UES participation entailed an expression of favorable qualities, where athletes themselves epitomized aspirational characteristics (13). These were recognized as positive, but equally associated with certain capitalistic trends (14), in which one must dominate the self to become a productive and contributing member of society (15). Through this guise, UEA can be seen as model citizens; that being individuals who can both overcome and achieve, standing out due to their superlative nature while blending into valued norms (12).

As western cultural ideologies became increasingly shaped by capitalist developments and resulting inequalities, the ability to maintain health came to be seen as admirable (16). Whereas historical applications of sport encompassed social correction and the encouragement of good behavior, in modern neoliberal environments exercise can be used to create a fit, athletic body as “proof” that one has overcome obstacles to achieve personal success (17). Though some comparisons can be made, neoliberal conceptions of health are distinguishable from collectivist social constructs in which group success and cohesion are central to goal achievement (18). For us, this distinction may explain why UES may be attractive in individualistic societies aligned with neoliberal models, where it is presumed that everyone has the same opportunity for success, but that only some will access it through individual resources and personal responsibility (19).

In contemporary settings that prioritize self-directed pursuits, sporting engagement does more than demonstrate discipline and motivation, but also can be seen to connect one's ability to value health with being perceived as a good citizen (12). Foucault has described comparable patterns, depicting humans as capable of dominating the machine of the body and gaining resulting power (20). Though Foucault developed his conceptions of power over time, he eventually believed in its productive, creative, and life-affirming characteristics (21). This power is portrayed as omnipresent, in everyone, and developed through the ability to have control over the body's function and processes (20). This may also be reciprocal, as the demonstration of bodily discipline through sport suggests that the person is acceptable, obedient, and also a good citizen (22). From this perspective, UES, where domination of the self is a required component of participation and often has the side-effect of creating a fit body, could be construed as conveying social value.

Accordingly, in the authors' opinion, if neoliberal systems praise self-directed fitness, it is difficult to imagine a more favorable activity than UES. Not only are athletes spending more time performing fitness, often training multiple hours and sometimes across several disciplines (as in triathlon), the nature of UES demands substantial motivation and perseverance. Further, the popularity of UES may be conditioned by a societal emphasis on physical activity in reaction to the obesity epidemic in the western world, which has had severe implications across medical, societal, and economic spheres (12, 19). Body shaming and weight bias can be significant issues in healthcare settings among practitioners (23). Resultantly, in certain related sociocultural frameworks, the public are exposed to messaging that stigmatizes higher body weight (23), linking this to a sedentary lifestyle, and underlines physical activity as a method for accessing wellbeing (24). Further, This can potentially engender a conditional (albeit contentious) logic that supports the idea of extreme physical activity in the form of UES (24). In broad societal representations, healthy individuals may be seen as personally responsible for this outcome, receiving accolades and approval for enhancing their wellbeing (25). For example, it is often assumed that weight loss constitutes an improvement in the self and is therefore celebrated within social groups and the media (26).

In western countries, governmental bodies and other applicable agencies have been eager to support this association, hosting running events as a method of encouraging citizens to counteract the effects of sedentary lives, poor diets (27), and declining mental health (12, 28). Notably, greater prominence may be placed on marketing ultra-distance sporting events with the aim of achieving trickle-down public health outcomes (29); this is demonstrated by ultramarathon events, which have seen an 1,676% increase in participation since 1996 (30). Analogously, researchers have argued that UES has assumed broader significance, becoming synonymous with improved morality (31). Elsewhere, others have contended that UEA might conceivably pursue an athletic challenge in UES that is designed to heighten the emotional experience and engender a sense of life-affirming pleasure (32). As Hanold suggests, the more challenging the athletic pursuit, the more it is valued, not just by the individual, but by society; through this link, individuals within communities can enjoy a certain prestige (33). In sum, UES may ostensibly encompass athletic pursuits, but in sociocultural contexts, participation represents more than just a game.

### 1.2. UES participation and health

Despite these associations, a question remains unaddressed across scientific literature: what is the specific relationship between UES participation and health? It has been posited that the human body has evolved to be successful in endurance sport (34), with roots in evolutionary genetics. Nevertheless, scientific evidence is inconclusive about the purpose and origins of physiological traits and the effect that bipedalism may have on the economy of movement (35). Additionally, the definition of “endurance” can be unclear in inquiries solely focused on running or walking, as these were the most available modes of ambulation when these characteristics emerged (35). These studies refer to running or walking interchangeably though they incur different impacts on human health due to the different demands that varying paces will have on the body (35). Current data showing the positive effects of physical activity often derive from moderate volumes (5, 36). Yet, it is unclear if the large volume of training required for UES yields greater health outcomes; instead, recent work has illustrated pathological risks for UEA (2, 37, 38). Despite the overwhelming evidence of the health benefits of exercise, we believe that, per the Yerkes-Dodson law, there may be a point in training volume beyond which adverse health outcomes may arise (39–41).

Many athletes pursue UES intending to attain, maintain, and improve their wellbeing through physical activity (42). Aside from this, UEA may gain other advantages from participation, such as an improved sense of self and social belonging (43, 44). In this regard, UEA can receive personal satisfaction and public approval (42), sentiments which are likely to encourage continued involvement. Yet, based on extant evidence, assessing whether UEA are physically and mentally healthy is challenging (45) owing to a lack of inquiry into the longitudinal effects of high-volume exercise and limited reporting of adverse incidents (46, 47). Thus, the pursuit of wellbeing may not necessarily constitute an essential component of UES participation for certain athletes, and these even can be divergent concepts even when the stated purpose is enjoyment. For instance, when confronted with physiological risks, some UEA can be galvanized and unwilling to stop, despite claiming to be highly oriented toward health-promoting behaviors (48).

Consequently, athletes may engage with and undertake harmful actions such as continuing to train despite injury or illness (49), restricting nutritional intake (49), and training in dangerous conditions (50). Though UES may revere such expressions of discipline and find them beneficial for competitive performance in specific races, disregarding somatic feedback can be detrimental (51). Notably, adverse effects from high volume training without adequate recovery, which is associated with UES, may include Overtraining Syndrome (OTS) (52, 53), Relative Energy Deficiency in Sport, and related endocrine dysfunction (54). High volume training could also lead to cardiac abnormalities (55), increased musculoskeletal injury (56), and elevated risks for mental illness (57). Moreover, while some UEA have acknowledged health as a motivating factor for UES participation (58), others reportedly participate for reasons that may be construed as opposing health, such as using the sport to support disordered eating habits or to enjoy resultant physical pain (42). Likewise, Kazimierczak and colleagues have described how UEA can derive fulfillment from purposely subjecting themselves to physical and mental pain (59); the opioid-like neurotransmitter response to endurance exercise may contribute as a significant motivator for this (60). Yet, the pursuit of pleasure from exercise may also conceivably have ill-effects comparable to those found in other behaviors that lead to addiction (61, 62). Importantly, exercise addiction (EA) is characterized by salience, tolerance, mood modification, withdrawal, and relapse, and can have severe consequences (61).

### 1.3. Study rationale

Against this background, we believe that UEA are people with powerful and heterogeneous motivations undertaking activities with underexplored physical and mental health effects. Whereas UES are generally deemed to be health-related and have positive associations in sociocultural frameworks, this may potentially contribute to contradictory experiences. For example, it may be true that one must engage in significant exercise to perform well in UES; nonetheless, as we have noted, data on the connection between high volume exercise and health remains limited. Likewise, if sociocultural pressures to avoid obesity and/or be perceived as successful compels a person to overtrain, this can have injurious effects. Consequently, more insight is needed to better understand the impact that these activities (in varying volumes) may have on UEA who are deeply aligned with their sport (48). In our view, these issues, coupled with the inconsistencies in the benefits of UES participation and growing public interest, require deeper insights into how these sports are represented in popular media, and how those portrayals align with scientific findings. Hence, this interdisciplinary study seeks to comprehend what aspects of UES are important for UEA participation (or those who intend to be athletes) and how these patterns intersect with wider sociocultural and health-related contexts of these activities.

## 2. Materials and methods

### 2.1. Introduction

To investigate our research question, we examined relevant YouTube videos on various UES to develop an understanding of what themes are encompassed by popular portrayals of the community; UES are comprehensively represented online, where UEA, or those hoping to become participants, can source informational materials (63). As a platform, YouTube was chosen because it is the second most accessed internet source and thus an influential archive of human culture (64). YouTube is diverse, global, and used by both amateur and professional creators to reach audiences (65). Users can watch as well and interact with information through liking, commenting, or reading others' comments, creating a powerful relationship *via* content engagement (65). One of the most important components of YouTube videos is authenticity (66, 67) and although a certain amount of creative license is expected (67), users may deem video content to be a reliable reflection of the subject matter's identity (68).

We adopted qualitative analysis techniques, such as the transcription and coding of spoken commentary, found in Namie and Warne's analysis of prominent themes gleaned from television commercials (69). We specifically applied this as a conceptual model to videos on various UES, including ultramarathon, endurance cycling, long distance triathlon, and endurance rowing (70).

### 2.2. Sampling and sample

On 16th January 2023, we conducted YouTube searches using the following terms for well-known UES: “ultramarathon running”, “ultra endurance cycling”, “Ironman triathlon”, “ultra endurance swimming”, and “ultra endurance rowing”. Subsequently, we selected the top three videos in each category filtered by relevance that had a duration over four minutes (this time period was predefined by YouTube selection options). We removed videos exclusively showing workouts without commentary, those containing bespoke additions (i.e., “Strongman Swimming”, which includes carrying a weighted object during the event), or those created to promote specific races. When one video was disqualified based on these criteria, we chose the next video in the list generated by the search. As we aimed to have an equal number of videos for each sport, the resulting sample (*n* = 15) included three videos per each sporting category. Videos ranged from 4:36 to 38:52 min in duration and all but one appeared to be professionally produced vs. vlog-style personal videos.

### 2.3. Data analysis

We used a multi-modal framework on this sample, enabling us to compare different types of videos (informational, instructive, interview) across the same theme to facilitate analysis of dynamic media (70). Conventional content analysis methods outlined by Hsieh and Shannon were adopted, in which the videos were not examined for presumed themes, but for those that organically emerged from the content (71). This allowed content to be scrutinized distinctly, which is effective in situations where discourse on a topic may be limited, thus facilitating the discovery of themes as presented without preconceived criteria. In our study, this included noting words and phrases from the videos, followed by a summative approach to connect spoken words to broader themes (71). Codes were then assigned to the themes that emerged and annotations were taken for where these appeared in the videos. This technique has been incorporated in research that seeks to understand the underlying meaning in situations where euphemisms may be utilized broadly by a specific group (71). We also employed content analysis methods as demonstrated by Wang and Feng in their study of TikTok videos, in which the most popular videos in a category are analyzed for thematic content, coded, and prominent themes are discussed (72). Per our research question, this enabled us to contextualize modern media depictions within a broader sociocultural framework (72).

### 2.4. Judgment criteria

We utilized the aforementioned checklist from Elo and colleagues to enhance trustworthiness and reliability in the preparation, organization, and reporting phases. Based on this, multiple members of the research team reviewed materials to corroborate identified interpretations of emerging themes (73), checking for overlap and accuracy. To ensure robust saturation, the equation for saturation ratio presented in Guest et al (74). was performed and can be found in Tables 1, 2 (2/5 = .45% saturation threshold).

**Table 1:** Base themes from first four videos.

Video #	1	2	3	4	Total
Base Themes	4	1	0	0	5

**Table 2:** Unique themes in all videos.

Video #	5	6	7	8	9	10	11	12	13	14	15	Total
New Themes	0	1	0	0	1	0	0	0	0	0	0	2

## 3. Results

After reviewing these *n* = 15 YouTube videos, we identified five prominent and recurring themes, as outlined below. A table showing a quantification of content themes is available in the supplemental materials.

*Theme 1*: The mental aspect of UES participation includes discussion of mental strength, mental toughness, mindsets, mental tricks, mental limits, obsessions, and addictions.

*Theme 2*: Some athletes share emotional stories of their UES experiences in a “folk-tale” style that are considered motivational.

*Theme 3*: There is great interest in what UEA eat to sustain long distances.

*Theme 4*: Pain and suffering are great sources of pride.

*Theme 5*: Words associated with mental illness as a colloquial descriptor of UES/UEA are often used to describe the sport, the people, and their habits.

Two additional themes were discerned, namely: references to nature and references to a feeling of community within UES. However, as neither repeated more than four times across the videos, they were not deemed to be significant and were not included in the analysis.

### 3.1. Themes in YouTube representations of UES

*The mental aspect of UES participation includes discussion of mental strength, mental toughness (MT), mindsets, mental tricks, mental limits, obsessions, and addictions*.

We observed this theme the most often (one hundred and nine times). Within the YouTube depictions, we identified a perception among UEA that the mind is as important as their physical body for successful participation. In this regard, ultra triathlon competitor Rich Roll describes what UES training signifies: “It’s the ultimate test of challenging the outer limits of my mental, emotional, physical, and spiritual capabilities.” (75). The hosts of a video on the psychological implications of UES have comparable views: “It’s a mental game as much as a physical one. Many people are physically capable of riding for ten hours, but it’s their head that holds them back […] your mind is your biggest asset.” (76). These hosts further reason that ultra endurance events are “75% mental, 25% physical.” (76).

Kevin Murphy, an ultra endurance swimmer, acknowledges that mentality was an essential attribute in his swimming: “I was never a great swimmer. I was never fast. I was never fit. But, I’m in the swimming hall of fame. Now how can that be? Because I had that willpower. That mental toughness. The ability to carry on.” (77). Ultramarathoner Courtney Dauwalter's husband emphasizes what he considers to be her mental control over instinct: “She’s wired a little differently. Something that Courtney has learned and something she’s gotten really good at is that when your body is telling you to stop, like, maybe you don’t need to stop.” (78).

Athletes also disclosed their relationship with adverse mental aspects and potential psychosocial issues, though notably these are still presented in a positive context. In ultrarunning, runner Tim Olson declares that he is “addicted” to his sport following prior experiences of a substance use disorder: “I was passionately addicted to drugs and I just needed to turn that passion toward something more healthy. So, switching over to […] ultrarunning was a way to heal my body, heal my mind.” (79). After breaking the women's record by two days at the Tour Divide (a two thousand seven hundred- and forty-five-mile bikepacking race), ultra endurance cyclist Lael Wilcox returned after two weeks to try for a better time: “I obsessed over it.” (80). Ultra endurance swimmer Adam Peruta concludes his TedX speech by encouraging viewers to look forward to any obstacles they may face, “I promise you–you will learn so much and discover so much that you never knew about yourself in those moments that truly make you uncomfortable.” (81).

*Some athletes share emotional stories of their UES experiences in a “folk-tale” style that are considered motivational*.

Another theme we identified in the videos on fifty-seven occasions is that athletes and those around them speak about their experiences in a romantic, folkloric style that seemingly minimizes the effort required to participate in UES while maximizing the allure. Throughout many of these video representations, there is an overt expression of emotion, a sense of grandiosity, and allusions to deep human experiences. For instance, when a UEA is asked how he developed an interest in ultrarunning, he recounts his first ultra distance run: “I walked out of a bar on my 30th birthday and decided that night I was going to run 30 miles […] At midnight I left the bar and […] I didn’t own running shorts […] I peeled off my pants […] and thought ‘Just run to Half Moon Bay’. It just felt right […] I made it 30 miles. It wasn’t pretty […] but it changed the course of my life.” (82). Ultra endurance rower Laura Try describes the “primitive existence” created by her cross-Atlantic rowing experience as something that “makes my brain feel so full and so healthy.” (83). Comparably, when Lael Wilcox recalls an experience riding over four hundred and eighty-two kilometers, she recounts her encounters with strangers, despite viable threats to her safety and her refusal to stop riding on a dangerous route (80).

Rich Roll explains his physical transformation from a self-described “couch potato” to an ultra endurance triathlete after switching to a plant-based diet: “I actually had to go outside to start exercising again just to burn off all of this excess energy that was buzzing inside my body. The pounds melted away seemingly effortlessly. I was getting stronger and faster with each successive week without even really trying.” (75). The hosts of a cycling show espouse similar sentiments when providing advice on ultra endurance cycling: “Your body can do whatever you want it to. It’s the mind that you need to convince.” (75).

One of Courtney Dauwalter's crew members praises her athletic ability: “She’s gotten a lot stronger because she works so hard. That’what I’ve seen more change, but […] the foundation of all that work is that she’s so strong.” (78). Finally, Kevin Murphy attempts to explain the deeper significance of UES: “People want to challenge their human frailties. I know I can beat the demons in my head that say, ‘You can’t do this.’” (77).

*There is great interest in what UEA eat to sustain long distances*.

Diet, food, and the quality of foods were alluded to fifty-three times. While some videos were produced to specifically discuss UEA nutrition strategies, almost all of them mentioned food intake or types of food. Tim Olson highlights his high fat paleo diet as integral to his success, noting that he likes to “eat really clean before a race.” (79). Furthermore, in the ultratriathlon video featuring Rich Roll, his vegan diet figures heavily. Roll contends that plants contain optimal nutrition for athletic performance and argues that the vegan diet “Will change who you are.” (75).

Nonetheless, per representations in the videos, healthy foods and healthy eating habits may not be ubiquitous among UEA. “I’m pretty big on ice cream,” says Lael Wilcox in response to a question as to what she enjoys eating after a race (80). Additionally, Wilcox is asked what “unconventional” foods she relies on intra-race: “whole milk” although it's “kind of gross […] really hard on the stomach, but great for your legs.” (80). During an ultramarathon event, Courtney Dauwalter struggles with nausea and vomiting, a situation that can threaten her ability to finish the competition. One of her crew members describes what food she eventually ate, “We had cheese quesadillas and we’re like, ‘Do you want to try some pancakes and syrup?’ and she said ‘Ooh! That sounds good.’” He later affirms, “She loves candy.” (78). David Spelman, an endurance rower who broke the world record in 2019 for a two-person kayak Atlantic Ocean crossing recounts his team's strategy: “The more calories we can ingest in a 24 h period, the better the performance will be” (84). Spelman also discussed taking in upwards of 8,000 calories per day mostly through “nut butters” (84).

*Pain and suffering are great sources of pride*.

Pain and references to suffering are mentioned thirty times in the videos. The YouTube content we analyzed suggests that the concept of “pushing through” is an accepted part of UES; based on the videos we investigated, pain is largely considered necessary, as is the purposeful ignorance of body signals—a theme we noted thirty times. This seemingly negative feature of UES is presented as an opportunity to demonstrate one's ability to endure and exhibit strength. Top ultramarathoner Courtney Dauwalter speaks about “getting into the pain cave” on several occasions when documenting her experiences at an ultramarathon event (78). This athlete affirms that a high school coach was instrumental in helping her become accustomed to physical suffering: “He taught me everything I know about how to go into the pain cave, that place where it really hurts, he was the one who taught us how to go in there and be ok with it.” (78). One of Dauwalter's crew members highlights the competitive advantages of this: “She has the ability to push through […] She went through a lot of pain and suffering at a young age and figured out that it’s not the end of the world.” (78).

Similarly, “As triathletes…we enjoy putting ourselves through pain,” says one of the hosts of Global Triathlon Network in a video about the most demanding triathlons (85). In another video, the ultramarathoner and author, Dean Karnazes indicates that the key to success is to: “Push through it. A lot comes down to grunt work. The important thing is that you keep pushing.” (82). On the topic of training for ultra endurance events, the professional cyclist, Laura Penhaul, describes the mental challenge of the sport, “It really strips you back to your raw behaviors. It strips you bare mentally. It takes you to the depths.” (86). Correspondingly, a sports psychologist explains the mindset of an UEA in a BBC video: “You know it’s going to hurt, but you’re willing to expose yourself to that. When they think about managing pain, they just love it. [The athlete must say] I might feel tired, I might feel uncomfortable, but I have to push through that.” (77). Finally, in a “how-to” video on training for ultra endurance events, the presenter argues that training should “teach your body and mind to keep going when already fatigued.” (86).

*Words associated with mental illness as a colloquial descriptor of UES/UEA are often used to describe the sport, the people, and their habits*.

As a frequent depiction occurring twenty-four times, the UEA in the videos we examined use phrases like “crazy”, “madness”, “weird”, and “psycho” to describe their participation in UES, the UEA community at large, and common practices associated with their sport. For example, in a video with over three and a half million views, the ultrarunner Tim Olson affirms: “To get into ultrarunning, you have to be crazy.” (79). Roman Möckli reasons similarly, “To row across the ocean, you need to be a little bit crazy.” (87). Analogously, David Spelman describes his teammate as a “fellow nutter.” (84). In outlining his plans to partake in English Channel crossings, the endurance swimmer, Peter Attia, says, “I went psycho and ratcheted up the training.” (88). Further, Dean Karnazes discussed his public attempt to break a Guinness World Record: “It’s crazy, it’s ludicrous to be saying this, but I’ve actually run for 48 h on a treadmill as well.” (82). Karnazes also recalls his first attempt at running over forty-two kilometers: “My friends thought I was crazy.” (82). Akin to this, Fox Sports Australia's *The Back Page* depicted Chloe McCardle's solo crossings of the English Channel as “madness.” (89). Adam Peruta, finisher of the Ultraman Triathlon, lists the many epithets he encountered when sharing his plans to compete in the five-hundred- and fifteen-kilometer race such as “crazy, dumb, idiotic, ridiculous, psychotic, not smart, moronic, laughable, and stupid.” (81).

## 4. Discussion

### 4.1. Sociocultural aspects of YouTube UES themes

Based on the themes we identified, the videos reveal important insights into the culture that supports and encourages UES participation. Although it is not possible to substantiate the utility of folklore-style anecdotes in conveying accurate information about UES, we do recognize their success as narrative devices in the videos we examined. From a sociocultural perspective, humans enjoy learning about each other through stories (90) and it has been suggested that we are more apt to share and take note of accounts that contain themes important to our evolutionary survival (91). While it is beyond the scope of this discussion to delve into the specific aspects of UES participation that could be considered complementary to these purposes, we believe that the hero's journey of human triumph over adversity is implicit in several of the narratives we reviewed in the YouTube videos (92). Accordingly, Campbell's hero's journey trope is, irrespective of topic, effective at creating a recognizable emotional arc, as well as drawing the viewer to personally invest in the story (93). Interestingly, echoes of many of Booker's Seven Basic Plots run throughout these videos, such as “Overcoming the Monster”, “The Quest”, “Voyage and Return”, “Comedy”, and “Tragedy”; all of which are engaging methods of storytelling designed to highlight self-actualization in the face of dramatic events (94). Taken together, these narratives are compelling because they focus on the hero at the center of the story and their ability to overcome obstacles—a recurring aspect of UES prevalent in our results (94).

In the authors' opinion, the athlete as “hero” depicted in these videos is the product of the society in which their demonstrated characteristics are valued. With current rates at 42% in the USA (95) and 26% in the UK (96), obesity is considered an important public health concern in modern western societies (97). In neoliberal contexts, a UEA's discipline and power demonstrate their control over their body and the resulting likelihood of preventing obesity (and ill-health), thereby benefiting society, culture, and the economy (98). Resultantly, those who perform fitness, live in thin and fit bodies, and display a commitment to health can gain approval, status, and value as a result (12, 22, 98). This portrayal may diminish the contribution of socioeconomic factors, education, the healthcare system, and even environmental issues (99), instead focusing on the individual who is uniquely able to attain good health through exercise. While we would not suggest that fighting against obesity is the central sociocultural, political, and economic concern of our time, it does appear that one's ability to avoid ill-health and therefore be a responsible citizen is often revered above other personal skills and attributes (100).

As the YouTube videos imply, the mental and physical challenge of UES suggest personal success overall and the athlete is foregrounded as the person who is capable of persevering through injurious circumstances. Yet, these conditions are not down to factors typical in hero narratives like war, disease, poverty, or natural disasters (92). They are also not due to overcoming systemic inequities and insufficiencies or lack of resources, as might characterize neoliberal systems (101). Instead, the UEA has chosen to put themselves through a test that has been created in the form of a sporting event, not one to which they have no choice but to submit. Moreover, the race environment may give the perception of personal control over adverse circumstances and a possibility of self-directed success through intra-race crisis management (25). For instance, resulting frustrations due to intra-race management scenarios were expressed by UEA in our results (78). Weedon discusses how similar activities were historically designed to combat “overcivilization” and that some extreme athletic events intend to continue in this vein (15).

This focus on pushing through pain and suffering may have wider, positive connotations in neoliberal settings, where this ability can be lauded by some individuals (12). Based on this, UES may be perceived to be one of the few remaining ways that a person can fully engage with suffering in a desirable way. Significantly, pain has been described as an integral part of running culture (102) and triathlon (103), which was alluded to in the videos we examined. Interestingly, several UEA in the videos described being “average” before their participation in UES (77, 79, 88), suggesting that the ability to “push through” could be equally or more important than traditional sporting ability. For us, the sense derived from using the term “average” in these situations is undesirable, seeming to confirm the idea that UEA find satisfaction in being different from others, which aligns with the importance of individual influences on health (101).

Interestingly, prior research has accentuated the pro-social benefits of UES (104). As the videos indicate, UES may be particularly compelling for participants due to the perceived benefits associated with personal investment in the sport. As previously highlighted, discussions around the community benefits of UES participation convey the aspirational quality of individuals who can successfully perform sport to pursue health (12, 19, 105). That said, only four of the *n* = 15 videos mentioned a sense of connectivity to others and two of these were specific to team rowing. UES are generally independent disciplines, though even singular participation ultimately leads to a sense of community allegiance (106). UEA can gain a strong sense of belonging and social credibility within the group, contributing to feelings of honor and satisfaction, among others (106). However, these benefits have also related to suffering; proving oneself as “tough enough” to perform UES can be valuable to upholding a sense of identity (106).

It is therefore understandable that in a culture that may be under pressure to be fit and healthy, those who can achieve such goals may conceivably gain a perceived elevated status, as was also noted in our findings. For example, one UEA discussed overcoming alcohol use disorder and a sedentary lifestyle, switching to a vegan diet and losing a substantial amount of weight as they became a successful, competitive triathlete (75). As previously discussed, there is a strong emphasis on physical activity in western societies, which may have both healthcare inadequacies and increasing rates of obesity (107, 108). These two issues together have accentuated the importance of fitness and have supported the idea that better health is dependent upon engaging in a certain kind of exercise regime (109). It has been argued that this cultural interest in fitness has created a false equivalency with accomplishment (25), and that self-directed pursuits of health are admirable (105). In such settings where athletes who conform to western society's dominant values such as strength, discipline, pain tolerance, and unrelenting drive, those who pursue “ultra” health through UES may be considered more generally successful on an individual level (19). Again, this was exemplified in the videos, where those who self-described as “addicts” or as experiencing apathy and ill-health, were able to find a sense of accomplishment through endurance exercise and accompanying dietary modifications (75, 79, 82).

### 4.2. YouTube UES themes and health

While the videos around UES participation be rooted in certain sociocultural issues, they appear to be inconsistent with current scientific evidence; traits that may result in accomplishments and accolades in certain contexts may not result in similarly positive outcomes in sporting contexts. Firstly, though the concept of mental toughness is represented in these videos as a source of pride, it does not always bring the desired results of discipline, commitment, and success in goal achievement (110, 111). Mental toughness is associated with traits that are fundamental to athletic success like the ability to persevere and remain focused (110), as well as human success in the display of power and strength (14). Contrastingly, it can simultaneously elicit potentially negative associations (111). There is a complex relationship between mental toughness, physical activity, and Dark Triad traits that are interrelated with psychopathy (110). Dark Triad characteristics may be effective for individual athletes, as narcissism can create an emphasis on personal achievements, Machiavellianism might encourage athletes to manipulate circumstances to ensure success, and psychopathy could engender an “anything it takes” philosophy in competitive circumstances (112). Nevertheless, Dark Triad traits can lead to violence towards fellow athletes, cheating behaviors, or use of performance enhancing drugs (113). More research is needed to understand mental toughness in UES, though based on initial analysis and its possible associations with negative behaviors, we do not believe that mental strength should be considered as an unquestionable positive trait, as was implied by the videos in our sample.

Acknowledging that the terms “crazy” and “psycho” are problematic and can engender increased stigma around mental health (114), it remains important to examine links between psychological issues and UES. Awareness about mental health in UEA is growing and preliminary insights may indicate concerning prevalence rates of mental disorder in this population (38, 115). Though phenomena of “craziness” and “quirky behavior” have been discussed in other explorations of UES (16), detailed inquiries are needed to corroborate these observations. In particular, it is important to better understand whether those with mental health conditions are particularly drawn to UES or if participation heightens psychological vulnerabilities (57). There is currently a lack of adequate scientific data on the potential mental health risks of UES participation, together with a limited understanding of whether sociocultural paradigms may increase applicable risk factors. We believe that the sparsity of scientific data is concerning, especially given the difficulty of presenting this evidence given extant cultural drivers; that is, we are concerned that even the most compelling correlations between mental illness and UES may do little to deter participation in a society that highly values co-occurring results.

Nevertheless, we contend that some of the language used in these videos may have psychopathological connotations, furthering associations between UES and mental health issues. Despite this, we do not believe that presence of a mental health condition implies that it is a prerequisite for participation. We are therefore left to hypothesize that perhaps the use of these colloquial terms might serve to set UEA apart from “normal” people by suggesting that they are “abnormal” (114), even though habits and behaviors in UES communities likely become normalized within those spaces (33). This could further exceptionalize UEA who may enjoy being different from other athlete groups (59). Yet, it is conceivable that many people may not see the colloquial usage of “crazy” as a synonym for “exceptional”, but instead consider it to be alienating and exacerbating mental health stigma among athletes (114).

An observed belief in UES communities is that ever-increasing volumes of exercise will bring about ever-increasing health and success (25). Concerningly, the link between mental health and exercise is also assumed to be dose dependent. Contrastingly, extant literature may imply that there is more likely a U-shaped curve between exercise and health (37, 38, 116). By focusing on anecdotes of running through the night or effortlessly changing body composition, risks for physical and mental injury become culturally minimized in these videos. This tendency does little to accurately educate individuals about assumed proper training techniques involving periodization, adequate rest, and supportive nutrition or how to avoid negative outcomes (4). Further, this fallacy perpetuates the myth that UEA can elevate themselves above adverse health consequences with additional training and may lead to serious injury (117).

Pushing through one's boundaries by ignoring both psychological and physiological distress to continue to train may lead to negative outcomes such as OTS (118). OTS is a serious condition that often affects athletes who have chronically neglected adequate rest and recovery (118). While this is not currently included in psychiatric diagnostic manuals, it is thoroughly discussed in medical literature (53). Whereas OTS risk factors are varied, they may include purposeful ignorance of fatigue, muscle soreness, and malaise (52, 53). As documented, several UEA have experienced OTS (105), some of whom were unable to return to participation, exemplifying how the notion of “pushing through” may entail detrimental health consequences. Correspondingly, development of EA may follow similar patterns of unhealthy training, as well as unrelenting behaviors and attitudes (117). Like OTS, exercise addiction is not included in current psychiatric diagnostic manuals, though it remains a prominent concern for endurance athletes (119).

Finally, the duration and intensity of UES events requires UEA to consume caloric supplementation intra-race and replenish depleted nutrition stores post-race. Environmental circumstances and digestion difficulties may mean UEA gravitate toward simple-digesting carbohydrates that are seemingly antithetical to health such as cookies, chips, candy, and soft drinks (120, 121), as was illustrated in the videos we investigated. Post-race, athletes discuss indulging in “treat” foods that are “unhealthy” (80). Food intake is a critical component of athletic success, though there is debate as to what type and quantity of food equates with success in UES (122).

It should be noted that we are unable to directly examine the nutritional needs of any specific UEA or the relative healthiness of their diet from these videos but there may be sizable popular interest in the quantity and types of food they eat. For us, a directorial choice could have depicted these athletes as eating low-quality food, contrasting with the cultural representation of dietary choices associated with health. Hence, it may be assumed that these athletes represent a paradox in that they are able to seemingly attain health while making nutritional choices that are in opposition to health. Despite this, it is unlikely that UEA do not count nutritional support among their resources, particularly since many are sponsored by intra-race nutritional supplements (123, 124). We therefore posit that this portrayal could be intended to make UEA seem more relatable, thus serving to humanize UES, which often appear to require superhuman abilities. From a marketing perspective, portraying the UEA lifestyle as achievable may be more impactful than suggesting that a complex nutrition protocol is necessary.

## 5. Conclusion

As interest in UES grows, we sought to develop an understanding of what aspects of participation are salient in the community and compelling to UEA and/or prospective UEA. We reviewed relevant YouTube videos depicting UES and extracted recurring themes to gain preliminary insights into what characteristics are valued in this community. We then compared these themes with extant literature and discussed their sociocultural context and possible discrepancies with scientific evidence. In the authors’ opinion, the videos we reviewed advance a compelling and persuasive argument for UES participation: UEA are portrayed as capable of impressive feats and overcoming physiological barriers while contending with potential dangers and injury. UES may allow an athlete to demonstrate their power to attain personal health despite the challenges of modern life, thereby cultivating a correlation with societal success. Based on our analysis, negative features of UES are construed as opportunities for personal gain and overcoming these is considered a normalized and necessary aspect of participation. As our results indicate, when coupled with a cultural predilection for fitness that can be evident in western society, the UEA is conveyed as a model of success, as representations imply that they are able to achieve their goals regardless of circumstance. For us, these associations may not align with all experiences of UEA and could even contravene accepted definitions of health. Certain themes presented in the videos could perpetuate unrealistic representations of UES which, if adhered to without context, may entail mental and physical consequences.

### 5.1. Questions for future study

Though our results highlight certain features of UES participation that may be important to UEA or aspiring athletes as depicted in the YouTube videos, broader questions about these activities endure, as outlined below. In the authors' view, the significant sociocultural complexities around UES and the underexamined health outcomes of participation underscore the need for further inquiry. Since the scope of these topics contain interdisciplinary intersections, we believe that information exchanges are necessary between various epistemological areas, involving researchers in sports medicine, sports psychiatry, and the social sciences.

#### 5.1.1. What other motivators underpin UES participation?

Per our results, the themes presented in the videos suggest that UES participation has implications beyond the scope of general fitness and physical health pursuits. Therefore, we believe further studies into the motivations that underpin UES are necessary, especially as these may not always be *prima facie* positive in origin; as an example, these could include qualitative interviews involving UEA and race organizers. Repeated allusions to self-inflicted pain (78) and anecdotes of placing oneself in potentially dangerous situations (80) cause us to consider the possible associations between these behaviors and more complex psychopathological frameworks (110). Additionally, we believe that it may be necessary to investigate the broader societal values in which this theme has been created to understand precisely how participation in UES is seen as positive and aspirational. It may follow that some UEA are an increasingly captive audience for commercial entities, as interest in the marketing and promotion of ultra distance races and athletic equipment grows. Advertising messages that are complementary to ideas of individual success and health could compel the athlete toward an unhealthy relationship with UES, as the desire to be perceived as successful theoretically supersedes respect for physiological limitations (19, 125).

#### 5.1.2. Is UES healthy?

As highlighted, findings on the relative healthiness of UES are, to the authors' knowledge, conflicting and not definitive (45–47). Prior research has assessed UEA health using sick days at work or school as a proxy (126), but we advocate for more detailed studies into the physical and mental considerations of UES for holistic wellbeing (127). For example, physiological and psychological health outcomes in those who engage in high volume training could be studied as a discrete cohort in order to avoid the assumption that health benefits exist equally across unequal measures of physical activity. Clarifying this would aid in diminishing the dissemination of potentially misleading information about UES, especially in cultural representations, and encourage athletes to consider practical training measures adapted to high volume training that would benefit longevity in the sport. In the authors' opinion, it is important to divide the cultural interest in this extreme form of physical activity from the realities of the limits of human physiology, particularly given the societal tendency to encourage exercise irrespective of amount (27). Correspondingly, we are concerned about potential biases in the overall representation of UES and a reluctance to consider the negative implications of participation. For this reason, we also support inquiry by parties both within and outside of the UES community.

#### 5.1.3. By whom and with whom should education be shared?

As our results highlight, the media plays a sizable role in the depiction of UEA and UES. We acknowledge that the athletes themselves are responsible for how they portray themselves in public forums and what information they choose to share. However, many may also be subject to contractual responsibilities as a result of sponsorship, thereby skewing or omitting information due to marketing obligations (128). In specific contexts, UEA are seen as role models and thought leaders in a genre of sport that has not benefited from extensive study; accordingly, their voice can be highly influential within their community and beyond (129, 130). Given the limited research on the health implications of UES and the lack of empirical data on associated practices that would preclude illness and injury, concerns arise that the sharing of information on these topics would fall upon anecdotal advice from certain UEA. To provide veracity and clarity, we advocate that governing bodies and stakeholders accurately examine the beneficial and detrimental outcomes of UES participation.

### 5.2. Limitations

Our study examined cultural representations of UES using *n* = 15 YouTube videos, offering insights into how these sports are currently represented to UEA and aspiring athletes. However, like many studies involving internet media, our research has several limitations (131). Firstly, the YouTube search was performed on a single day (16th January 2023). Due to the large amount of content uploaded to YouTube, we may have omitted pertinent, newer videos. Content was selected according to its relevance since these would be the top videos that the average user would find when searching these topics. Owing to proprietary YouTube algorithms, alongside variations in how content creators identify a video through tagging, repetitive searches may not reveal the same search results. Nonetheless, we believe that our sample includes relevant examples of popular media representations of this community.

The videos we reviewed may have been produced with the goal of presenting interesting content and thus could have been edited to dramatize these themes. As Sakib and colleagues posit, videos edited in this way are commonly found in health-conscious media (129); in the authors' opinion, our research into YouTube portrayals of UES suggest that this is the same in social media representations of these events. However, we believe that it is important to analyze the videos as they are presented, just as any end-consumer would, with the intention of receiving education and possibly comparing themselves to the representations they are watching. This is because consumers of YouTube videos are likely watching with the intent to know more about the topic, to gain understanding of UES, and potentially to learn how to become a UEA themselves (132). As previously mentioned, YouTube content remains highly influential (64), though it is often not subject to academic rigor. Accordingly, we recognize that directorial choices may influence the content presented and we cannot assume that it fully represents the entire UES community. It should be noted that all UEA featured in videos were white, and while it is not possible to verify educational, employment, or socioeconomic status for all athletes, they are depicted as capable of purchasing technical sporting equipment, living in desirable locations, and enjoying leisure activities. We acknowledge that racial, educational, and socioeconomic factors are wide-ranging and may thus differ from presented content.

UES share many important characteristics that differentiate them from other sporting activities, but they remain heterogeneous within their category. Therefore, certain themes may be more naturally associated with one sport, but not with others. For example, the benefits of being out in nature were discussed in some of the videos (78, 79, 83), but not all UES involve training in nature. Swimming is often performed indoors, rowers may use an indoor ergometer rowing machine, and runners and cyclists can use treadmills or stationary bikes. Likewise, some sports require relatively small financial outlay and others could require large investments for equipment or even fund-raising to be able to participate, such as the cross-Atlantic Talisker Challenge (133). Therefore, as mentioned previously, it is likely that cultural micro-differences may exist contingent on the applicable activity and community, which would require further nuanced inquiry. Despite this, we believe that our methodology provides preliminary insights into holistic cultural representations of UES and how these relate to scientific findings. As we were able to achieve a robust saturation rate (.4%), we feel that the themes in our results adequately reflect beliefs currently circulating in the community.

### 5.3. Concluding observations

As scholarly research does not fully align with assertions within the content we explored, this raises concerns about health-related risks in the UES community. Features of UES are clearly attractive, as exemplified by the view counts in our results, but it is unclear if they are always attainable. Therefore, participating individuals, governments and other organizational bodies should be provided with information that clarifies the relationship between UES and health, given the inclination to encourage and support ultra endurance athletic events in public spaces. Hence, in the authors' opinion, increasing interest in UES warrants an increasing responsibility to present accurate information to the public, through extensive research and knowledge sharing.

## Data Availability

The original contributions presented in the study are included in the article/**Supplementary Material**, further inquiries can be directed to the corresponding author.

## Footnotes

^1^For clarity, it should be noted that the word “athlete” is used throughout this paper to describe any individual who trains for and/or competes in sporting events, although we acknowledge that this term may carry connotations that inadvertently suggest improved health.
