# Supplementary material for: Ultra culture-ultra reality: a content analysis of YouTube depictions of ultra endurance sport and comparisons to scientific literature
Source: Front Sports Act Living. 2023 Jul 25;5:1192401. doi: 10.3389/fspor.2023.1192401 (PMC10411528; doi:10.3389/fspor.2023.1192401)
Supplement: Supplementary file 1 [file Table1.docx]

Supplementary Table 1: Quantified theme analysis per video

| Video | Sport | Views | Run Time | Theme 1 | Theme 2 | Theme 3 | Theme 4 | Theme 5 |
| --- | --- | --- | --- | --- | --- | --- | --- | --- |
| Ultramarathoner Answers Questions- (82) | Ultramarathon | 1.4M | 14:59 | 5 | 6 | 4 | 2 | 2 |
| Courtney Dauwalter-The Source(78) | Ultramarathon | 2.5M | 38:52 | 26 | 7 | 6 | 6 | 1 |
| High Fat Diet of Ultrarunner-Munchies(79) | Ultramarathon | 3.5M | 12:57 | 8 | 5 | 11 | 1 | 2 |
| 21 Questions-Lael Wilcox(80) | Ultra Cycling | 130k | 12:36 | 3 | 5 | 4 | 2 | 2 |
| Train for Ultra Endurance-GCN(76) | Ultra Cycling | 316k | 6:08 | 7 | 1 | 2 | 1 |  |
| 6 Golden Rules-GCN(86) | Ultra Cycling | 443k | 16:04 | 6 |  | 5 | 1 | 1 |
| Joe Rogan-Ultra Swimmers(88) | Ultra Swimming | 3.8M | 11:38 | 5 | 4 |  | 2 | 4 |
| Chloe McCardle-The Back Page(89) | Ultra Swimming | 742 | 8:32 | 6 | 2 | 2 | 1 | 3 |
| Psychology of Extreme Endurance-BBC Reel(77) | Ultra Swimming | 15k | 4:36 | 13 | 2 |  | 5 | 1 |
| 7 Toughest Triathlons-GTN(85) | Triathlon | 81k | 7:20 | 1 | 4 |  | 1 | 1 |
| Ultra Endurance Athlete-Rich Roll(75) | Triathlon | 102k | 5:59 | 5 | 8 | 6 |  |  |
| Ultraman Triathlon-Adam Peruta, TedX(81) | Triathlon | 80k | 16:49 | 9 | 3 |  | 5 | 4 |
| Resilient Adventures-Dave Spelman(84) | Ultra Rowing | 879 | 18:59 | 8 | 3 | 5 | 2 | 2 |
| World's Toughest Row-Roman Möckli, TedX(87) | Ultra Rowing | 1.6k | 9:06 | 2 | 1 | 3 |  | 1 |
| Rowing 3000 miles across the Atlantic Ocean-Laura Try(83) | Ultra Rowing | 4.4M | 20:43 | 5 | 3 | 5 | 1 |  |
| Total | - | - | - | 109 | 57 | 53 | 30 | 24 |
